# Supplementary material for: Development of a computer-aided design software for the quantitative evaluation of aesthetic damage
Source: PLoS One. 2019 Dec 18;14(12):e0226322. doi: 10.1371/journal.pone.0226322 (PMC6919621; doi:10.1371/journal.pone.0226322)
Supplement: S1 Code site — 1. (ZIP) [file pone.0226322.s001.zip › [EESC_jr][Projeto]Relatorio_07_21/reports/templates_zip/gentelella-master/vendors/google-code-prettify/CHANGES.html]

Change Log

README

# Known Issues

- Perl formatting is really crappy. Partly because the author is lazy and
  partly because Perl is
  hard to parse.- On some browsers, `<code>` elements with newlines in the text
    which use CSS to specify `white-space:pre` will have the newlines
    improperly stripped if the element is not attached to the document at the time
    the stripping is done. Also, on IE 6, all newlines will be stripped from
    `<code>` elements because of the way IE6 produces
    `innerHTML`. Workaround: use `<pre>` for code with
    newlines.

# Change Log

## 29 March 2007

- Added tests for PHP support
  to address
  issue 3.- Fixed
    bug: `prettyPrintOne` was not halting. This was not
    reachable through the normal entry point.- Fixed
      bug: recursing into a script block or PHP tag that was not properly
      closed would not silently drop the content.
      (test)- Fixed
        bug: was eating tabs
        (test)- Fixed entity handling so that the caveat
          > Caveats: please properly escape less-thans. x&lt;y
          > instead of x<y, and use " instead of
          > &quot; for string delimiters.

          is no longer applicable.- Added noisefree's C#
            patch- Added a distribution that has comments and
              whitespace removed to reduce download size from 45.5kB to 12.8kB.

## 4 Jul 2008

- Added language specific formatters that are triggered by the presence
  of a `lang-<language-file-extension>`
- Fixed bug: python handling of `'''string'''`- Fixed bug: `/` in regex `[charsets] should not end regex`

## 5 Jul 2008

- Defined language extensions for Lisp and Lua

## 14 Jul 2008

- Language handlers for F#, OCAML, SQL- Support for `nocode` spans to allow embedding of line
    numbers and code annotations which should not be styled or otherwise
    affect the tokenization of prettified code.
    See the issue 22
    testcase.

## 6 Jan 2009

- Language handlers for Visual Basic, Haskell, CSS, and WikiText
- Added .mxml extension to the markup style handler for
  Flex MXML files. See
  issue 37.- Added .m extension to the C style handler so that Objective
    C source files properly highlight. See
    issue 58.- Changed HTML lexer to use the same embedded source mechanism as the
      wiki language handler, and changed to use the registered
      CSS handler for STYLE element content.

## 21 May 2009

- Rewrote to improve performance on large files.
  See benchmarks.
- Fixed bugs with highlighting of Haskell line comments, Lisp
  number literals, Lua strings, C preprocessor directives,
  newlines in Wiki code on Windows, and newlines in IE6.

## 14 August 2009

- Fixed prettifying of `<code>` blocks with embedded newlines.

## 3 October 2009

- Fixed prettifying of XML/HTML tags that contain uppercase letters.

## 19 July 2010

- Added support for line numbers. Bug
  22
- Added YAML support. Bug
  123
- Added VHDL support courtesy Le Poussin.
- IE performance improvements. Bug
  102 courtesy jacobly.
- A variety of markup formatting fixes courtesy smain and thezbyg.
- Fixed copy and paste in IE[678].- Changed output to use `&#160;` instead of
    `&nbsp;` so that the output works when embedded in XML.
    Bug
    108.

## 7 September 2010

- Added support for coffeescript courtesy Cezary Bartoszuk.

## 4 March 2011

- Added a themes
  gallery to showcase contributed styles.
- Added support for XQuery courtesy Patrick Wied, Nemerle
  courtesy Zimin A.V., and Latex support courtesy Martin S.

## 29 March 2011

- Fixed IE newline issues, and copying/pasting of prettified
  source code from IE. This required significant internal changes
  but involves no API changes.
  **Caveat:** `prettyPrintOne` injects the HTML
  passed to it into a `<pre>` element.
  If the HTML comes from a trusted source, this may allow XSS.
  Do not do this. This should not be a problem for existing apps
  since the standard usage is to rewrite the HTML and then inject
  it, so anyone doing that with untrusted HTML already has an XSS
  vulnerability. If you sanitize and prettify HTML from an
  untrusted source, sanitize first.

## 4 February 2013

- Language handlers for Dart, Erlang, Mumps, TCL, R, S., and others
- Bug fix: VB REM style comments.
- Bug fix: CSS color literals / ID selector confusion.
- Bug fix: IE8 line breaks.

## 24 February 2013

- Added a one script autoload&run mechanism and a way to
  embed hints in processing instructions/comments.
  See example.

## 4 March 2013

- Matlab language handler courtesy Amro³
